# CC Chemokines in Myocardial Fibrosis: Regulatory Networks of CCL17 and Emerging Therapeutic Implications

**DOI:** 10.3390/ijms27083458

**Published:** 2026-04-12

**Authors:** Weiting Cai, Jing Zhao, Zheng Zhang

**Affiliations:** 1 Department of Coronary Care Unit, The First Hospital of Lanzhou University, Lanzhou 730000, China; caiccu@163.com; 2 Heart Center, The First Hospital of Lanzhou University, Lanzhou 730000, China; ldyy_jingzhao@lzu.edu.cn

**Keywords:** CC chemokines, myocardial fibrosis, CCL17, regulatory T cells, cardiac remodeling, heart failure

## Abstract

Myocardial fibrosis is a key pathological process driving the progression of cardiovascular diseases toward heart failure, closely linked to persistent inflammation and immune dysregulation. Among CC chemokines, CCL17 has emerged as an important mediator connecting immune cell dynamics with fibrotic remodeling. This review outlines current understanding of the cellular sources, regulatory mechanisms, and functional roles of CCL17, with particular attention to its impact on regulatory T cell (Treg) recruitment through ligand-biased signaling. Beyond this mechanism, CCL17 likely operates within a broader inflammatory network, with potential interactions involving CCR2^+^ macrophages and IL-17-related pathways. Experimental studies show that disruption of CCL17 signaling attenuates fibrosis and improves cardiac function, while clinical data link elevated circulating CCL17 to cardiac dysfunction and adverse outcomes. However, the absence of clinical trials and the redundancy of chemokine networks remain key challenges for translation. Overall, CCL17 may serve as a biomarker and therapeutic target, although its clinical application will require a more integrated, network-based understanding.

## 1. Introduction

Myocardial fibrosis represents a common pathological substrate underlying the progression of diverse cardiovascular diseases toward heart failure. It is characterized by excessive activation and proliferation of cardiac fibroblasts together with abnormal accumulation of extracellular matrix components, particularly collagen, ultimately leading to increased myocardial stiffness, electrical conduction disturbances, and impaired contractile function [1,2]. Following cardiac injury such as myocardial infarction, fibrotic remodeling initially serves as an adaptive repair response that preserves ventricular wall integrity and facilitates tissue healing. However, persistent and excessive matrix deposition results in scar expansion beyond the infarcted region, progressively contributing to cardiac dysfunction [2]. Increasing evidence suggests that injury-driven reparative fibrosis constitutes a major component of pathological remodeling, with immune-mediated inflammation acting as a central driver of disease progression [3].

After myocardial injury, dying cardiomyocytes release damage-associated molecular patterns (DAMPs), which rapidly initiate a cascade of immune responses involving the recruitment and activation of neutrophils, monocytes/macrophages, and lymphocytes [4]. These immune cells orchestrate inflammatory signaling through the secretion of cytokines and chemokines, thereby shaping both the magnitude and duration of the inflammatory response and ultimately influencing fibrotic remodeling outcomes [3,5]. Within this complex regulatory network, chemokines play a pivotal role by directing immune cell trafficking and functional differentiation through interactions with specific receptors [6,7]. Among them, CC chemokines have emerged as key mediators linking inflammation to fibrosis.

Recent studies have drawn attention to CCL17, a CC chemokine predominantly produced by specific immune cell subsets, including CCR2-positive macrophages and dendritic cells. Elevated CCL17 expression has been observed during aging and under cardiac stress conditions and is closely associated with adverse cardiac remodeling [8]. Mechanistic investigations further indicate that CCL17 regulates myocardial inflammation through biased signaling mechanisms that limit the recruitment of cardioprotective regulatory T cells (Tregs). Interestingly, while CCL17 deficiency enhances Treg accumulation in viral myocarditis models, it may simultaneously impair viral clearance, suggesting context-dependent immunomodulatory effects. These findings highlight the complex and environment-dependent role of CCL17 within the cardiac immune microenvironment. Therefore, a comprehensive understanding of CCL17 regulatory networks and downstream signaling pathways is essential for elucidating the immunological basis of myocardial fibrosis and identifying potential therapeutic strategies. This review is based on a narrative synthesis of recent literature retrieved from databases including PubMed and Web of Science, using keywords such as “CCL17”, “myocardial fibrosis”, and “cardiac remodeling”.

## 2. Overview of the CC Chemokine Family and Its Role in Cardiovascular Disease

### 2.1. Structural and Functional Characteristics of CC Chemokines

CC chemokines constitute a subgroup of cytokines defined by the presence of two adjacent cysteine residues near the N-terminus of their protein sequence, a structural feature that forms the basis of their classification and nomenclature [9]. These ligands exert their biological functions primarily through interaction with G protein-coupled receptors (GPCRs), which represent a specialized branch within the broader GPCR superfamily involved in immune signaling [9]. For instance, human CCL7 has been shown to promote coupling of chemokine receptors to heterotrimeric G proteins and β-arrestins, thereby initiating canonical GPCR signaling cascades [9].

Despite sequence diversity, CC chemokines generally adopt conserved three-dimensional folds that support receptor recognition and signaling activity. Structural studies demonstrate that CCL7 maintains a stable conformation in solution; however, interspecies variations can introduce functional diversity. An example is murine CCL2, whose highly glycosylated and structurally disordered C-terminal region alters both overall folding and glycosaminoglycan-binding properties, illustrating how evolutionary divergence shapes chemokine specificity and function [10].

Functionally, CC chemokines primarily regulate leukocyte trafficking and activation, thereby contributing to inflammatory responses, immune surveillance, and tissue homeostasis [11]. Within the cardiovascular system, they coordinate immune cell recruitment following tissue injury and influence downstream inflammatory cascades. Similar regulatory roles have been observed in other organs; for example, members of the macrophage inflammatory protein-1 family (CCL3, CCL4, and CCL9) and their receptors CCR1 and CCR5 are markedly upregulated after traumatic brain injury and participate in neuroinflammatory processes [12]. Beyond inflammation, CC chemokines also contribute to bone homeostasis through regulation of osteoclasts, osteoblasts, and progenitor populations [11].

A defining feature of chemokine biology is the promiscuous ligand–receptor interaction network, in which multiple ligands engage overlapping receptor sets. This organization provides functional redundancy while preserving signaling specificity, yet it also complicates selective pharmacological targeting [13]. Structural analyses indicate that conserved aromatic residue clusters located within the second extracellular loop (ECL2) and the upper regions of transmembrane helices 4 and 5 enable receptors such as CCR1, CCR2, and CCR5 to discriminate among closely related ligands [13]. Such refined molecular recognition ensures precise regulation of immune cell migration under diverse physiological and pathological conditions.

### 2.2. Dual Roles of CC Chemokines in Inflammatory Injury and Tissue Repair

During the early phase of tissue injury, such as acute myocardial infarction, CC chemokines are rapidly upregulated and orchestrate the recruitment of monocytes and other inflammatory cells to damaged sites, facilitating clearance of necrotic debris and initiation of tissue repair. Among these mediators, monocyte chemoattractant protein-1 (MCP-1/CCL2) has been extensively studied and consistently shows increased expression across diverse inflammatory and injury models [14,15]. Accordingly, controlled modulation of chemokine signaling has been proposed as a strategy to limit excessive inflammation and delay fibrotic progression. For example, in a traumatic brain injury model, the anti-inflammatory effects of sitagliptin were partly attributed to reduced MCP-1 expression in the striatum, leading to attenuation of local inflammation [14]. However, accumulating evidence suggests that chemokine function is highly context dependent. In a trichloroethylene-induced immune kidney injury model, complement component C5a regulated MCP-1 expression and exerted stage-specific protective effects during tissue injury [15].

Similar bidirectional effects have been observed in cardiovascular disease. Under conditions such as pressure overload or aging-associated cardiomyopathy, sustained chemokine expression promotes chronic low-grade inflammation characterized by macrophage and lymphocyte infiltration, thereby enhancing fibroblast activation and collagen deposition. Loss of CCL2 has been shown to alter immune cell differentiation through activation of the MST1–mTORC1–STAT1 signaling axis, reshaping B-cell populations and suggesting a broader immunoregulatory role beyond monocyte recruitment [16]. In atherosclerosis models, the natural compound kaempferol suppresses MCP-1 expression in THP-1-derived macrophages and inhibits monocyte migration, further supporting the contribution of MCP-1/CCL2 signaling to chronic vascular inflammation and fibrosis [17].

Increasingly, CC chemokines are recognized as context-dependent regulators of tissue remodeling, capable of exerting either pro-fibrotic or anti-fibrotic effects depending on temporal expression patterns, receptor availability, and downstream signaling activation. Time-dependent expression of CCL2, CCL4, and CCL5 during luteal regression in pigs illustrates their complex roles in apoptosis and steroidogenic regulation [18]. Similarly, CCL4 may promote tumor progression by recruiting regulatory immune populations while also enhancing antitumor immunity through cytotoxic lymphocyte recruitment under specific conditions [19]. CCL7 exhibits comparable duality within tumor microenvironments by modulating immune cell trafficking and stromal responses [20]. Collectively, these observations highlight the dynamic and environment-dependent nature of chemokine signaling, suggesting that balanced chemokine networks are essential for effective cardiac repair, whereas their dysregulation may shift healing toward pathological fibrosis.

## 3. Regulation of CCL17 Expression and Cellular Sources in Myocardial Fibrosis

### 3.1. Inducers of CCL17 Expression and Identification of Cellular Sources

Multiple experimental models of cardiac injury consistently demonstrate robust induction of CCL17 expression within cardiac tissue. Elevated CCL17 levels have been observed in myocardial ischemia–reperfusion injury, chronic angiotensin II (Ang II) combined with phenylephrine (PE) infusion, and diphtheria toxin-mediated cardiomyocyte ablation models [21]. These findings suggest that CCL17 represents a common stress-responsive chemokine generated during diverse forms of cardiac injury. In addition to local cardiac expression, circulating CCL17 concentrations increase with advancing age and correlate positively with the severity of cardiac dysfunction [22]. Analyses derived from community-based cohorts and heart failure populations further indicate that serum CCL17 levels rise progressively with aging and are closely associated with impaired cardiac performance, supporting its potential value as a biomarker of age-related cardiac remodeling and pathological functional decline [22].

The cellular origin of CCL17 in the injured heart has been clarified through flow cytometric analyses combined with genetic knockout models. Following cardiac injury, infiltrating CCR2^+^ macrophages and CD11b^+^ classical dendritic cells (cDCs) have been identified as the principal producers of CCL17 [21]. These immune cell populations represent key effector components of the inflammatory response, and their activation, together with subsequent CCL17 secretion, contributes to shaping the post-injury cardiac inflammatory microenvironment. In both myocardial infarction and Ang II/PE infusion models, CCR2^+^ macrophages and CD11b^+^ cDCs constitute the dominant cellular sources of myocardial CCL17 expression [21]. Establishing this cellular origin directly links CCL17 production to defined immune subsets and provides an essential framework for understanding its immunoregulatory role in myocardial fibrosis.

### 3.2. Key Signaling Pathways Regulating CCL17 Expression

Transcriptomic analyses have revealed that CCL17^+^ macrophages and dendritic cells display distinct gene expression signatures, identifying granulocyte–macrophage colony-stimulating factor (GM-CSF) signaling as a major upstream regulator of CCL17 expression [21]. Mechanistically, GM-CSF promotes coordinated activation of STAT5 and canonical NF-κB pathways, which together drive transcription of the CCL17 gene. This cooperative signaling between STAT5 and NF-κB is considered a central mechanism sustaining elevated CCL17 expression under cardiac stress conditions [21].

Evidence from other disease contexts further supports a broader regulatory role for NF-κB signaling in CCL17 induction. In airway epithelial cells infected with human rhinovirus, activation of NF-κB markedly enhances CCL17 expression [23]. Similarly, in atopic dermatitis models, stimulation with TNF-α and IFN-γ increases CCL17 production through combined activation of NF-κB and STAT1 pathways [24]. Additional regulation has been linked to MAPK signaling, where p38 MAPK and JNK pathways contribute to CCL17 expression in keratinocytes [25]. Although multiple signaling cascades participate in its regulation, the GM-CSF–STAT5/NF-κB axis appears to play a dominant role in cardiac immune cells. Understanding this pathway provides a potential framework for transcriptional intervention, as targeting GM-CSF or its downstream STAT5 and NF-κB signaling components may limit pathological CCL17 upregulation.

Beyond transcriptional control, the cytokine microenvironment also shapes CCL17 expression. During IL-4-induced polarization of macrophages toward an M2 phenotype, MEK5/ERK5 signaling enhances CCL17 production through regulation of c-Myc expression [26]. Under hypoxic conditions, macrophage polarization toward an M2-like state is accompanied by increased expression of CCL17 and related cytokines, a process associated with VEGF–HIF-1α signaling pathways [27]. Collectively, these findings indicate that CCL17 expression is governed by an integrated and dynamically regulated signaling network influenced by both intracellular pathways and extracellular immune cues.

## 4. Core Mechanism by Which CCL17 Aggravates Myocardial Fibrosis: Impaired Treg Recruitment

### 4.1. Biased Signaling of the CCL17–CCR4 Axis

CL17 exerts its biological effects primarily through CCR4, but this interaction does not elicit uniform downstream signaling. Instead, CCL17 induces ligand-biased activation of CCR4, leading to signaling outputs distinct from those triggered by its homologous ligand CCL22 [21]. Although both ligands share the same receptor, their downstream pathways diverge, with CCL17 preferentially activating Gq-dependent signaling while attenuating β-arrestin-mediated responses [21,28]. Given that β-arrestin signaling is closely associated with receptor internalization and regulatory T cell (Treg) migration, this selective bias limits Treg recruitment and shifts the local immune environment toward a pro-inflammatory state.

In the setting of myocardial injury, this signaling divergence translates into impaired immune resolution. By counteracting CCL22-driven Treg chemotaxis, CCL17 promotes sustained inflammation and fibrotic remodeling, linking receptor-level bias to functional outcomes in cardiac injury [21].

Recent evidence further expands the receptor landscape of CCL17. In addition to CCR4, CCL17 can engage CCR8 as an alternative high-affinity receptor, mediating immunoregulatory effects independent of canonical CCR4 signaling [29]. Through this pathway, CCL17 suppresses Treg activity in a CCL3-dependent manner even in the absence of CCR4, suggesting a broader and more flexible signaling network. These findings indicate that CCL17 operates within a multi-receptor system and imply that targeting CCR4 alone may be insufficient to fully modulate its downstream effects.

### 4.2. Competitive Inhibition of Treg Recruitment by CCL17 in the Injured Myocardium

The functional consequence of CCL17 signaling in the injured myocardium is most clearly reflected in its ability to restrain regulatory T cell (Treg) recruitment. By limiting β-arrestin-dependent signaling downstream of CCR4, CCL17 counteracts CCL22-driven Treg chemotaxis, resulting in reduced local immunoregulatory capacity and a shift toward sustained inflammation [21].

This mechanism is supported by loss-of-function studies. CCl17-deficient mice consistently exhibit increased myocardial Treg accumulation following cardiac injury, accompanied by attenuation of adverse remodeling [21]. Specifically, genetic deletion of CCl17 reduces myocardial fibrosis, limits cardiomyocyte hypertrophy, and improves left ventricular systolic function in models of myocardial infarction and angiotensin II-induced stress [21]. Quantitative analyses further indicate a 30–50% reduction in fibrotic area together with significant improvement in left ventricular ejection fraction, underscoring the functional impact of this pathway [21,22].

Collectively, these findings position CCL17 as a key regulator that links biased chemokine signaling to impaired immune resolution. By constraining Treg recruitment, CCL17 sustains inflammation and promotes fibrotic remodeling, thereby providing a mechanistic basis for targeted intervention.

## 5. Therapeutic Strategies Targeting CCL17 in Myocardial Fibrosis

### 5.1. Protective Effects Demonstrated in Genetic Knockout Models

CCL17 has emerged as an important pro-inflammatory regulator during pathological cardiac remodeling following myocardial injury. Evidence derived from genetic loss-of-function models provides direct support for its contribution to adverse remodeling. In both myocardial infarction and pressure overload models induced by angiotensin II (Ang II) combined with phenylephrine (PE), mice lacking CCl17 exhibit markedly improved cardiac structure and function compared with wild-type controls [21]. Specifically, CCl17-deficient animals display reduced left ventricular dilation, diminished myocardial fibrosis, attenuated cardiomyocyte hypertrophy, and enhanced systolic performance [21]. These structural and functional improvements collectively indicate that loss of CCL17 mitigates the progression of pathological cardiac remodeling.

Mechanistic analyses further demonstrate that these protective effects are closely associated with alterations in the post-injury immune microenvironment. Injured myocardium from CCl17 knockout mice shows reduced levels of pro-inflammatory cytokines together with a substantial increase in regulatory T cells (Tregs), a population known to suppress excessive immune activation and limit fibrotic responses [21]. CCL17 promotes biased signaling through CCR4 by preferentially activating Gq-dependent pathways while competitively interfering with β-arrestin signaling initiated by CCL22, thereby restricting Treg recruitment to damaged cardiac tissue [21]. In the absence of CCL17, this inhibitory constraint is relieved, allowing enhanced Treg homing and improved immune regulation.

Taken together, genetic evidence consistently identifies CCL17 as a driver of maladaptive cardiac remodeling, at least in part through limiting cardioprotective Treg infiltration. These findings provide a strong mechanistic rationale for targeting CCL17 signaling as a potential therapeutic strategy to attenuate myocardial fibrosis.

### 5.2. Translational Potential of Neutralizing Antibody Therapy

Although genetic knockout studies have provided important mechanistic insight into the pathogenic role of CCL17, clinical translation ultimately requires pharmacologically actionable strategies. Targeting CCL17 using neutralizing antibodies has therefore emerged as a promising therapeutic approach with direct translational relevance. Experimental evidence demonstrates that administration of anti-CCL17 neutralizing antibodies partially recapitulates the protective phenotype observed in genetic deletion models, significantly attenuating angiotensin II (Ang II)-induced pathological cardiac remodeling [21]. By specifically binding circulating and tissue-derived CCL17, these antibodies prevent interaction with the CCR4 receptor and thereby disrupt downstream signaling activation.

Consistent with findings in CCl17-deficient mice, antibody-mediated inhibition enhances recruitment of regulatory T cells (Tregs) to the myocardium, accompanied by reduced inflammatory responses and attenuation of fibrotic remodeling [21]. Importantly, these results indicate that transient pharmacological suppression of CCL17 activity can achieve cardioprotective effects comparable to permanent genetic modification, providing strong preclinical support for therapeutic targeting of this pathway.

Future therapeutic strategies may extend beyond neutralizing antibodies to include monoclonal antibodies, small-molecule CCR4 antagonists, and ligand-trapping fusion proteins. Notably, specific macrophage subsets, such as CD206^+^ IL-4Rα^+^ macrophages, have been implicated in fibrosis and adverse remodeling during ischemic cardiomyopathy and heart failure [30], while CCL17 is predominantly produced by CCR2^+^ macrophages and dendritic cells [21]. These observations suggest that targeting CCL17 may simultaneously modulate macrophage function and T cell-mediated immune regulation, thereby exerting broader effects on the fibrotic network. Collectively, anti-CCL17 therapy represents a potential immunomodulatory strategy aimed at reshaping the cardiac immune microenvironment to slow disease progression.

## 6. Perspectives and Future Directions in CCL17 Research

### 6.1. Integration of Current Evidence and Clinical Implications

Current evidence supports a model in which CCL17 functions as an upstream regulator of the post-injury immune microenvironment, linking CCR2^+^ inflammatory myeloid cells with impaired regulatory T cell (Treg)-mediated resolution pathways [21]. Rather than acting as an isolated chemokine, CCL17 occupies a nodal position within the inflammatory network that governs the transition from adaptive repair to maladaptive remodeling. Experimental studies consistently show that disruption of CCL17 signaling enhances myocardial Treg accumulation and attenuates fibrotic remodeling, supporting its role in sustaining inflammation and structural deterioration [21]. Beyond this axis, CCL17 is likely integrated within a broader inflammatory network, with potential crosstalk involving IL-17-associated pathways and CCR2^+^ macrophage activation, pointing to a coordinated immune–metabolic regulatory framework.

Evidence from human cohorts further supports the clinical relevance of this pathway. Elevated circulating CCL17 levels are associated with impaired cardiac function and an increased risk of adverse cardiovascular outcomes [31,32]. Circulating CCL17 also rises with aging and correlates with cardiac dysfunction [22]. In hypertrophic cardiomyopathy, increased CCL17 in the context of clonal hematopoiesis is linked to a higher risk of major adverse cardiovascular events [31]. Although these findings remain associative, they suggest that CCL17 may serve as a biomarker of immune activation and disease susceptibility.

From a therapeutic perspective, both genetic deletion and antibody-mediated inhibition of CCL17 attenuate cardiac hypertrophy and fibrosis in experimental models [22], supporting its potential as an immunomodulatory target. Currently, no clinical trials specifically targeting CCL17 in cardiovascular disease have been reported. However, circulating CCL17 has emerged as a potential biomarker associated with aging-related cardiac dysfunction and adverse cardiovascular outcomes [31,32]. However, no clinical trials specifically targeting CCL17 in cardiovascular disease have been reported to date. Moreover, given the redundancy of chemokine networks, whether selective inhibition of CCL17 alone is sufficient to achieve sustained clinical benefit remains uncertain. Future studies should therefore clarify its role within broader inflammatory circuits and define its value in patient stratification and targeted therapy. A summary of the key mechanisms and evidence is provided in Table 1.

### 6.2. Outstanding Questions and Future Research Directions

Despite growing experimental evidence, the relevance of CCL17 in human cardiac fibrosis remains incompletely defined. Current data are largely limited to circulating biomarkers or selected patient populations, and whether CCL17 independently predicts disease progression requires validation in large, well-characterized cohorts [22,32]. Future studies should further define its spatiotemporal expression in both myocardial tissue and circulation across different heart failure phenotypes.

Beyond its effects on regulatory T cells (Tregs), the broader cellular targets of CCL17 remain unclear. CCR4 is expressed on multiple immune cell subsets, including Th2 cells and basophils, whose roles in cardiac remodeling are not fully understood [21]. In addition, chemokines may directly regulate cardiac resident cells; for instance, CXCL12 promotes fibroblast activation, whereas CXCL10 suppresses fibroblast migration and fibrosis [6]. Whether CCL17 directly modulates fibroblast function and extracellular matrix production warrants further investigation.

From a translational perspective, therapeutic targeting of the CCL17 axis is still at an early stage. Although genetic deletion and antibody-mediated inhibition show beneficial effects in preclinical models [21,22], clinical translation faces several challenges. Chemokine networks are highly redundant, and inhibition of a single pathway may trigger compensatory activation of parallel axes such as CCL2/CCR2 and CCL5/CCR5 [6,33]. Moreover, no clinical trials targeting CCL17 in cardiovascular disease have been reported to date. Safety also remains a concern, as CCR4 is broadly expressed across immune cell populations, and systemic inhibition may disrupt immune homeostasis or impair host defense [34]. These limitations suggest that more selective or context-dependent approaches will be required. Future studies should therefore address not only efficacy but also long-term safety and integration with existing therapeutic strategies [4].

## 7. Conclusions

Current evidence positions CCL17 as a central immunoregulatory node linking inflammatory activation to impaired resolution in myocardial fibrosis. By modulating immune cell dynamics at the interface of innate and adaptive immunity, CCL17 sustains inflammation and promotes maladaptive cardiac remodeling, in part through restricting regulatory T cell (Treg)-mediated repair processes.

Experimental studies consistently demonstrate that genetic deletion or pharmacological inhibition of CCL17 attenuates fibrosis and improves cardiac function, supporting its potential as a therapeutic target. However, current evidence remains largely derived from preclinical models, and the relevance of CCL17 signaling in human disease—particularly across different etiologies and disease stages—has yet to be fully established.

Future work should therefore focus on validating CCL17 as a biomarker and therapeutic target in clinical settings, while accounting for the complexity and redundancy of chemokine networks. A more integrated understanding of CCL17 within broader inflammatory and immune–metabolic circuits will be essential for translating mechanistic insights into effective and safe therapeutic strategies.

## Figures and Tables

**Table 1:** Summary of the role of CCL17 in myocardial fibrosis: mechanisms, evidence, and therapeutic implications.

Category	Key Findings	Implications	References
**Cellular sources**	CCL17 is primarily produced by CCR2^+^ macrophages and CD11b^+^ dendritic cells in injured myocardium	Links innate immune activation to downstream adaptive immune regulation	[21]
**Core signaling mechanism**	CCL17 activates CCR4 with ligand-biased signaling, favoring Gq pathways while attenuating β-arrestin signaling	Limits regulatory T cell (Treg) recruitment and impairs immune resolution	[21,28]
**Functional consequence**	Reduced Treg infiltration leads to sustained inflammation, enhanced fibrosis, and adverse cardiac remodeling	Establishes CCL17 as a pro-inflammatory driver in cardiac injury	[21]
**Non-canonical pathway**	CCL17 can signal through CCR8 as an alternative receptor, suppressing Treg activity via a CCL3-dependent mechanism	Expands the signaling network beyond CCR4 and increases mechanistic complexity	[32]
**Interaction with inflammatory networks**	Potential crosstalk with CCR2^+^ macrophages and IL-17–associated pathways	Suggests integration within a broader immune–metabolic regulatory axis	[5]
**Preclinical evidence**	Ccl17 deficiency reduces fibrosis (~30–50%), limits hypertrophy, and improves cardiac function	Supports therapeutic targeting of the CCL17 axis	[21,22]
**Clinical evidence**	Elevated circulating CCL17 is associated with cardiac dysfunction and adverse cardiovascular outcomes	Indicates potential role as a biomarker in human disease	[30,31]
**Therapeutic potential**	Neutralization or genetic deletion of CCL17 attenuates cardiac remodeling in experimental models	Promising immunomodulatory target	[22]
**Limitations and challenges**	No clinical trials available; chemokine redundancy (e.g., CCL2/CCR2, CCL5/CCR5) may compensate	Single-target strategies may be insufficient	[5,33]
**Safety considerations**	CCR4 is broadly expressed across immune cells (Tregs, Th2, basophils)	Risk of immune imbalance and impaired host defense	[34]
**Future directions**	Need for human validation, selective targeting strategies, and integration with existing therapies	Emphasis on network-based and translational approaches	[4]

## Data Availability

No new data were created or analyzed in this study.

## Abbreviations

Ang II	angiotensin II
CCR2	CC chemokine receptor 2
CCR4	CC chemokine receptor 4
CCR8	CC chemokine receptor 8
CCL17	CC chemokine ligand 17
CCL22	CC chemokine ligand 22
CXCL10	C-X-C motif chemokine ligand 10
CXCL12	C-X-C motif chemokine ligand 12
ECM	extracellular matrix
HF	heart failure
IL-17	interleukin-17
LVEF	left ventricular ejection fraction
MI	myocardial infarction
Treg	regulatory T cell
